# HYPONASTIC LEAVES 1 is required for proper establishment of auxin gradient in apical hooks

**DOI:** 10.1093/plphys/kiab455

**Published:** 2021-10-02

**Authors:** Paula Vacs, Rodolfo Rasia, Nahuel González-Schain

**Affiliations:** Instituto de Biología Molecular y Celular de Rosario, CONICET, Facultad de Ciencias Bioquímicas y Farmacéuticas, Universidad Nacional de Rosario, Rosario, Argentina

## Abstract

During seedling germination under the soil surface, HYPONASTIC LEAVES 1 regulates apical hook development by modulating the formation of an auxin gradient.

Dear Editor,

Seedlings germinating under the soil surface have evolved an exquisite developmental program termed skotomorphogenesis. In darkness, dicot seedlings rapidly increase the hypocotyl length toward the surface in search of light, while protecting the apical meristem against mechanical damage by forming a hook between the hypocotyl and the two closed cotyledons (Josse and Halliday, 2008). A proper skotomorphogenic growth must be achieved until seedlings reach the light to ensure survival as they depend on limited seed reserves. Thus, plant development in darkness is tightly regulated by a complex network of transcription factors, phytohormones, and several signaling molecules involved in different biochemical and cellular processes (Gommers and Monte, 2018, Mazzella et al., 2014). We recently reported that microRNA (miRNA) biogenesis is necessary for proper skotomorphogenic growth in *Arabidopsis thaliana* (Sacnun et al., 2020). By studying mutants in the core components of the miRNA microprocessor, such as DICER LIKE 1 (DCL1), HYPONASTIC LEAVES 1 (HYL1), and SERRATE (SE), we showed that hypocotyl elongation in the dark requires all these proteins, probably through the action of specific miRNAs. Surprisingly, we found a microprocessor-independent function of HYL1 as a repressor of hook development. *hyl1-2* mutants failed to form and/or maintain the hook at early growth stages, while *dcl1 and se* mutants displayed a delayed hook unfolding. Together with other findings, we suggested a repressive role of the phosphorylated form of HYL1 in hook opening through the control of the activity and stability of the master regulator of photomorphogenesis ELONGATED HYPOCOTYL 5 (HY5). However, how HYL1 influences differential growth in hooks is still an open question.

The first report of a *HYL1* mutant showed altered sensitivity to exogenous auxins in roots from light-grown seedlings, and contrasting effects with auxin transport inhibitors (Lu and Fedoroff, 2000). Changes in auxin sensitivity can be attributed to an imbalance of several steps in auxin biology, from biosynthesis and perception to transport, signaling, or transcriptional reprogramming. As auxins were shown to be key players in hook formation and maintenance (Abbas et al., 2013) we then hypothesized that *hyl1-2* phenotypes displayed during skotomorphogenesis might be linked to affected auxin biology. *hyl1-2* knock-out mutants are unable to complete the apical hook formation phase and/or the maintenance phase is too short, resulting in more open hooks than in WT during skotomorphogenic growth (Sacnun et al., 2020 and Figure 1A). Several lines of evidence point to the phosphorylated form of HYL1 as a repressor of hook opening, including *se-1* mutants where HYL1 is unable to be dephosphorylated by C-TERMINAL DOMAIN PHOSPHATASE-LIKE 1, CPL1 (Manavella et al., 2012) explaining a prolonged maintenance phase in hooks. As auxins are essential for hook formation and maintenance (Abbas et al., 2013) we hypothesized that hook defects displayed in *HYL1* mutants might be linked to limited auxin levels. Hence, we tested the ability of *hyl1-2* to restore hook defects by applying exogenous indole acetic acid (IAA). Results shown in Figure 1A indicate that increasing amounts of exogenous auxins cannot rescue hook formation in *hyl1-2* as no statistical differences were found between treatments during skotomorphogenic growth (Supplemental Table S1). Even with supraoptimal amounts of auxins that affect WT hook development (1 µM IAA, Supplemental Figure S1) *hyl1-2* hooks failed to form. Since apical hook formation could be restored in auxin biosynthesis double mutants *wei8-3 tar2-1* (*weak ethylene insensitive8-3 tryptophan aminotransferase related2-1*) treated with 0.5 µM IAA (Cao et al., 2019), our results rule out the possibility that *hyl1-2* hook defects are due to reduced auxin biosynthesis or levels. However, an excess of auxins can also hinder hook formation as shown for WT in Supplemental Figure S1 or in plants that overproduce IAA, like *sur1* mutants (Boerjan et al., 1995) or YUC1 overexpressors (Zhao et al., 2001). We then tested the expression levels of auxin biosynthesis genes in *hyl1-2* and *se-1* mutants during skotomorphogenesis by RT-qPCR as an indicator of auxin levels. We chose the most expressed auxin biosynthesis genes in darkness according to a recent work with GUS reporter lines (Brumos et al., 2020), the two tryptophan aminotransferases *TAA1 and TAR2*, and two flavin monooxygenases, *YUC4 and YUC6*. These results show no clear indication of a consistent altered production of enzymes involved in auxin biosynthesis (Figure 1B).

**Figure 1:**
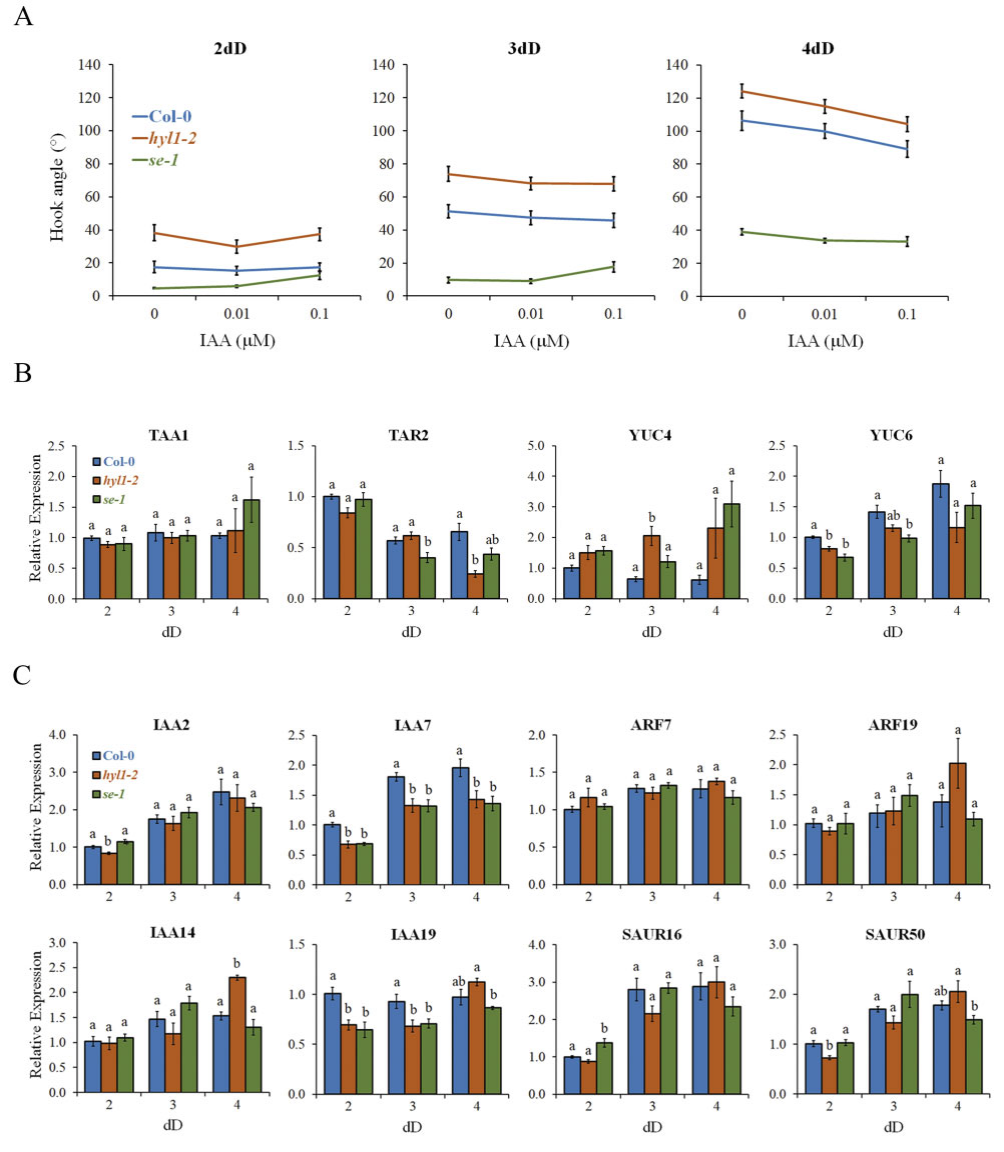
Affected hook development in *HYL1 and SE* mutants does not seem to be due to impaired auxin biosynthesis or misregulation of auxin-responsive genes. A, Hook angle measurements of 2-, 3-, and 4-d dark-grown (dD) at 22°C Col-0, *hyl1-2* (SALK_064863), and *se-1* (CS3257) seedlings. Both mutants are in Col-0 genetic background. IAA was added to the growth medium (0.5×MS) to the final concentrations indicated, where ethanol was added to the final concentration of 0.0008% for the mock treatment (0 µM IAA). Hook angle (the angle between the hypocotyl and an imaginary line between the cotyledons) was measured with ImageJ 1.49k software. Data are reported as mean ± SEM of between 21 and 64 seedlings from two biological replicates. No statistical differences were found between treatments during skotomorphogenic growth (Table S1). B and C, RT-qPCR analyses of Col-0, *hyl1-2, and se-1* dark-grown seedlings at the indicated time points. Primers used for auxin biosynthesis genes (B) and auxin-responsive genes (C) are listed in Table S2. Expression levels of indicated genes were normalized to the *PP2AA3* housekeeping gene and expressed relative to the Col-0 2dD value set at unity. Means ± SEM is shown from technical triplicates and two biological replicates. Statistically significant differences between groups are indicated by different letters (one-way ANOVA and Tukey’s post-test, *P* < 0.05). Statistical analysis was carried out with Infostat Software v. 2017 (Facultad de Ciencias Agropecuarias, Universidad Nacional de Córdoba, Córdoba, Argentina).

We next tested whether *hyl1-2 and se-1* hook phenotypes were due to affected auxin transcriptional responses by performing expression analyses of several auxin-responsive genes. We chose two auxin response factors (*ARF7 and ARF19*), four *Aux/IAA* (*IAA2*, *IAA7*, *IAA14*, and *IAA19*), and two small auxin-up RNA (*SAUR16 and SAUR50*) genes to study their expression levels by RT-qPCR during skotomorphogenesis. Most of these genes are necessary for correct seedling development in darkness (Sun et al., 2016, Zadnikova et al., 2010, Tatematsu et al., 2004). Only expression levels of *IAA7 and IAA19* genes were consistently downregulated in both mutants compared to WT in early skotomorphogenesis, although these differences were rather small (Figure 1C). With a few exceptions in specific time points, the remaining genes show similar expression levels to the WT. Altogether we found no trend in auxin production or transcriptional misregulation of auxin-responsive genes in *HYL1* or *SE* mutants that could be associated with the differences observed in hook development.

Polar auxin transport (PAT) is essential for correct hook development, where auxin efflux carriers of the PIN family play a prevalent role in the generation of auxin maxima in the concave side of the hook (Zadnikova et al., 2010). Interestingly, developmental defects in hooks of single *pin3*, *pin4*, *pin7*, and double mutants phenocopy those of *hyl1-2*. Naphthylphtalamic acid (NPA) is a well-known auxin efflux inhibitor that impedes hook formation (Teale and Palme, 2018). We tested how mutant hooks quantitatively respond to NPA by applying a lower than usual concentration (<5 µM) to study a possible dose-dependent effect. Figure 2A shows that while excess NPA hinder hook formation in all genotypes, undersaturating NPA levels does so with different intensity depending on the genotype (Supplemental Table S1). The slopes observed between 0.005 µM and 0.05 µM show that the NPA response of *hyl1-2* null mutant is weaker than WT while *se-1* displayed the opposite phenotype, in line with the hook developmental defects observed previously. As *se-1* is a hypomorphic allele of *SE* we confirmed this response, together with the IAA response, in *se-2* mutants (Supplemental Figure S2). NPA responses at 0.05 µM (Figure 2A) seem to be close to saturation, thus we narrowed the window of NPA concentration and tested at 2dD and 3dD. Results shown in Supplemental Figure S3 indicate that the minimum NPA concentration to produce an effect in hooks remains unchanged between genotypes, that is, sensitivity to NPA is not affected. However, the strength of NPA response is clearly different and opposite in microprocessor mutants compared to WT (Figure 2A and Supplemental Figure S3), probably due to auxin transport carriers, the NPA targets, being affected. We interpreted that PAT might be affected in microprocessor mutants so we decided to test it by other means. We introduced the transcriptional auxin reporter DR5 (Ulmasov et al., 1997) into the *hyl1-2 and se-1* mutants by genetic crosses. We first verified that the DR5-GUS construct does not affect the physiological behavior in *hyl1-2* or *se-1* backgrounds (Supplemental Figure S4). GUS assay was performed in all three genotypes in a time-course manner during skotomorphogenesis and, as expected, blue signals in the upper parts of the seedlings are observed mainly in cotyledons and hooks (Figure 2B). Inspired by a previous work (Béziat et al., 2017) we next quantified auxin response levels in the middle part (the bisector) of the hook (Figure 2C), using ImageJ to convert the photographed GUS staining to grayscale and measuring gray intensities (detailed in legends of Figure 2D). Strikingly, the auxin gradient in *hyl1-2* hooks is compromised during early skotomorphogenesis, the effect being more evident at 2dD and 3dD where auxin function is more relevant for hook development (Figure 2D). This result is in line with its weaker response to NPA and its inability to form the hook. *se-1* mutants, on the contrary, show an altered distribution of auxin marker toward the concave side of the hook at 2dD (Figure 2D). This hyperpolarization of auxin marker in *SE* mutants is also in line with its stronger response to NPA and its prolonged hook maintenance phase. To quantitatively evaluate this phenotype, we calculated the total area intensity along the hook (Supplemental Figure S5B) and the relative distance that corresponds to half of the total signal intensity, as an arbitrary measurement of auxin response distribution (Auxin Response Distribution Index [ARDI], Supplemental Figures S5A and S5C). Total integrated signals in *hyl1-2* were consistent and significantly lower than in WT background during early skotomorphogenesis (Supplemental Figure S5B). Also, the ARDI tends to be displaced to the central part of the hook in *hyl1-2* mutants, although it is statistically significant at 3dD only (Supplemental Figure S5C). Auxin gradient in *se-1* mutants is only significantly affected at 2dD, with lower integrated GUS levels (Supplemental Figure S5B) and an ARDI displaced to the concave side of the hook (Supplemental Figure S5C).

**Figure 2:**
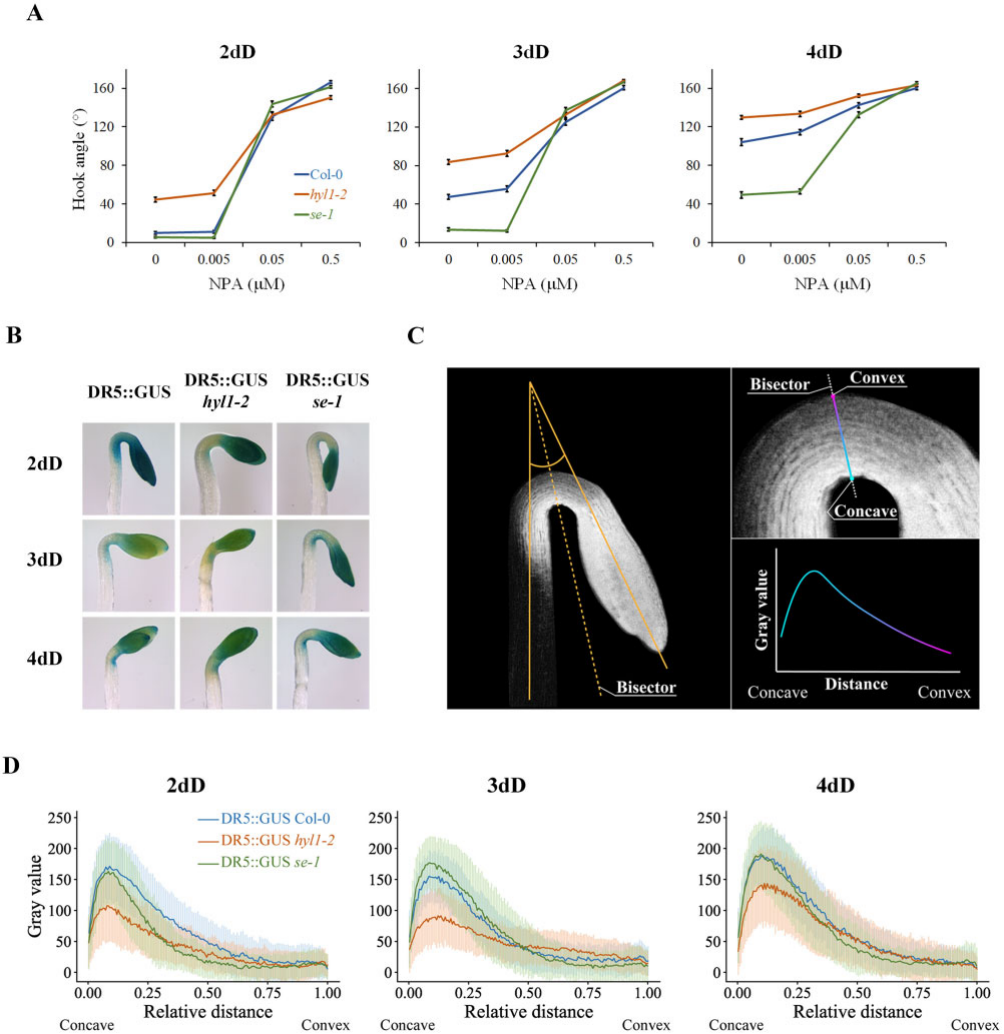
HYL1 is necessary for auxin gradient formation in hooks. A, NPA response assay in hooks of 2-, 3-, and 4-d dark-grown (dD) at 22°C Col-0, *hyl1-2*, and *se-1* seedlings. The PAT inhibitor NPA was added to the growth medium (0.5×MS) to the final concentrations indicated. Hook angles values were measured with ImageJ 1.49k software and are represented as mean ± SEM of between 31 and 73 seedlings from two biological replicates. Statistical significance between genotypes and treatments in all three time points were found (two-way ANOVA, Tukey’s post-test, *P* < 0.05, Table S1). B, Representative dark-grown DR5::GUS, DR5::GUS *hyl1-2*, and DR5::GUS *se-1* seedlings at the indicated time points, fixed 20 min at RT with 90% acetone and then stained with 2 mM X-Gluc (5-bromo-4-chloro-3-indolyl-beta-D-glucuronic acid) for 2 h at 37°C. Marker lines were obtained by crossing microprocessor mutants with DR5::GUS, which are all in Col-0 genetic background. C, scheme showing the chosen bisector of hook angle to measure the auxin gradient as gray values (GUS-blue signals were first transformed to gray signals with ImageJ 1.49k software) along the hook, between concave and convex sides. D, Gray value pattern of GUS signals along the hook of 2-, 3-, and 4-d dark-grown (dD) at 22°C DR5::GUS, DR5::GUS *hyl1-2*, and DR5::GUS *se-1* seedlings were measured with ImageJ 1.49k software. As hook diameters are not homogenous among seedlings we normalized measured sections from zero (concave part) to one (convex part) and next we calculated the mean and SD at each point (intervals) from each genotype (*n* = between 10 and 38 for each sample, with three biological replicates) by using a home-made script in Python (https://github.com/paulavacs/GUSquantification.git). Gray values (in arbitrary units) are represented as mean ± SD (Dark-colored lines and light-colored bars, respectively). Statistical analysis was carried out with Infostat Software v. 2017 (Facultad de Ciencias Agropecuarias, Universidad Nacional de Córdoba, Córdoba, Argentina).

Our results indicate that HYL1 is necessary to promote the auxin gradient in hooks, which is essential to maintain the apical meristem protected until seedlings reach the light. The behavior of *se-1* hooks from this work, together with the proposed role of SE as a scaffold for HYL1 dephosphorylation by CPL1 (Manavella et al., 2012, Sacnun et al., 2020) point to the phosphorylated form of HYL1 as an important player in auxin-mediated differential growth in skotomorphogenesis. It remains to be determined whether HYL1 specific action is carried out through its influence in the expression, localization or levels of auxin transport carriers.

## Accession numbers

Sequence data from this article can be found in the GenBank/EMBL data libraries under accession numbers HYL1 (At1g09700) and SE (At2g27100).

## Supplemental data 

The following materials are available in the online version of this article.

**Supplemental Figure S1.** Supraoptimal levels of IAA affect growth rate.

**Supplemental Figure S2.** Physiological behavior of *se-2* mutants in darkness is similar to *se-1*.

**Supplemental Figure S3.** NPA response assay in hooks of microprocessor mutants.

**Supplemental Figure S4.** Physiological behavior of DR5::GUS marker lines in darkness.

**Supplemental Figure S5.** Auxin differential accumulation in *hyl1-2 and se-1* hooks.

**Supplemental Table S1.** Statistical analysis (Figure 1A and Figure 2A).

**Supplemental Table S2.** Primers used in qRT-PCR (Figure 1B and Figure 1C).

## Supplementary Material

kiab455_Supplementary_Data

## Notes

N.G.-S. designed the study and wrote the article. P.V. and N.G.-S. performed the experiments. P.V., R.R., and N.G.-S. analyzed the data and revised the manuscript.

The author responsible for distribution of materials integral to the findings presented in this article in accordance with the policy described in the Instructions for Authors (https://academic.oup.com/plphys/pages/general-instructions) is Nahuel González-Schain: (schain@ibr-conicet.gov.ar).
