# Supplementary material for: HYPONASTIC LEAVES 1 is required for proper establishment of auxin gradient in apical hooks
Source: Plant Physiol. 2021 Oct 2;187(4):2356–60. doi: 10.1093/plphys/kiab455 (PMC8644733; doi:10.1093/plphys/kiab455)
Supplement: kiab455_Supplementary_Data [file kiab455_supplementary_data.pdf]

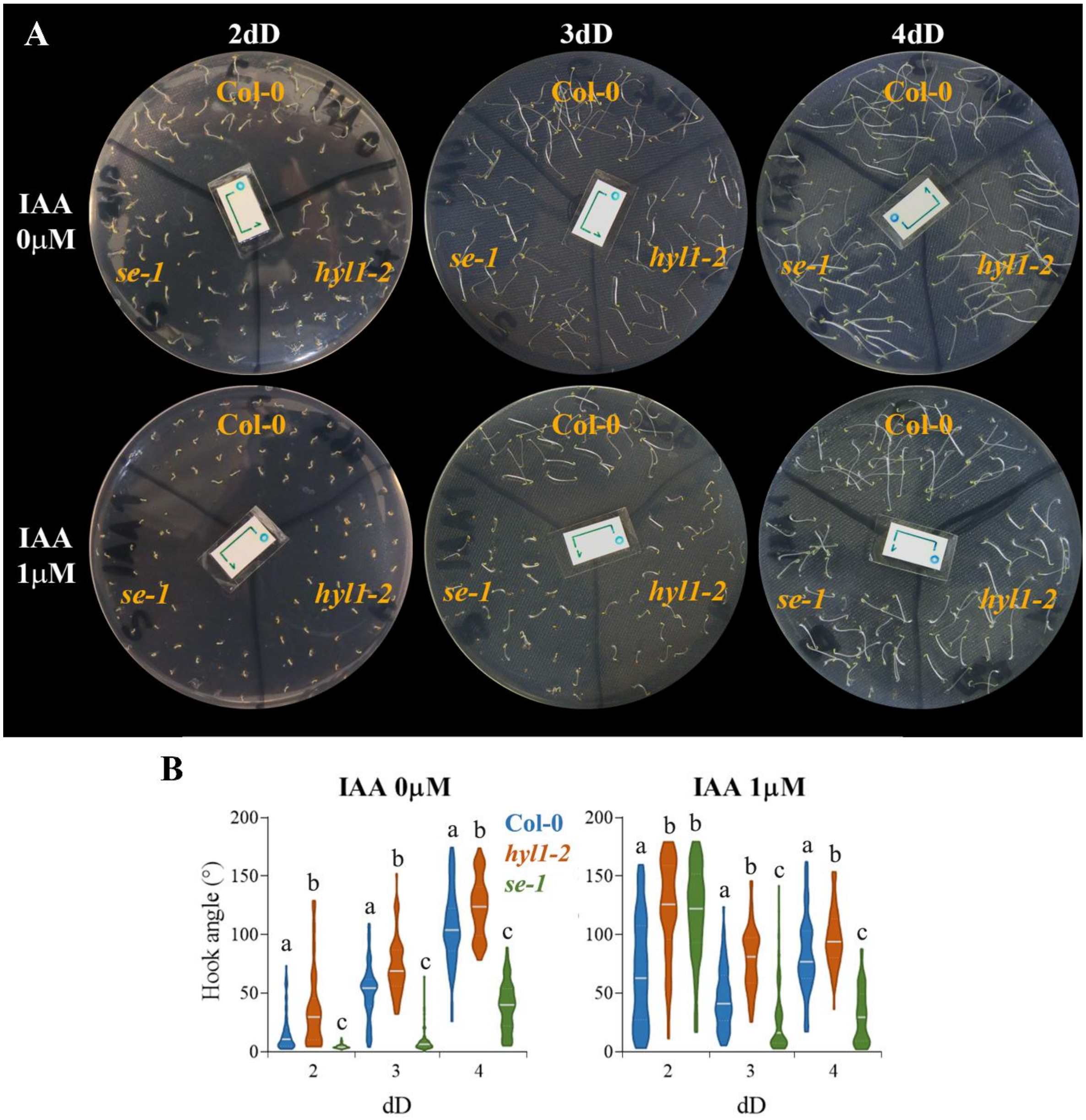

Supplemental Figure S1. Supraoptimal levels of IAA affects growth rate. (A), 2, 3 and 4-days dark-grown (dD) at 22°C Col-0, *hyl1-2* and *se-1* seedlings in 0.5xMS treated with 1μM IAA or 0.0008% ethanol (mock) as final concentrations. Seedlings were gently laid down in the medium before taking the photographs. (B), hook angle measurements of seedlings from (A). Between 21 and 36 seedlings for each sample and two biological replicates were measured. Statistically significant differences between groups are indicated by different letters (ANOVA and Tukey's post-test, $p < 0.05$).

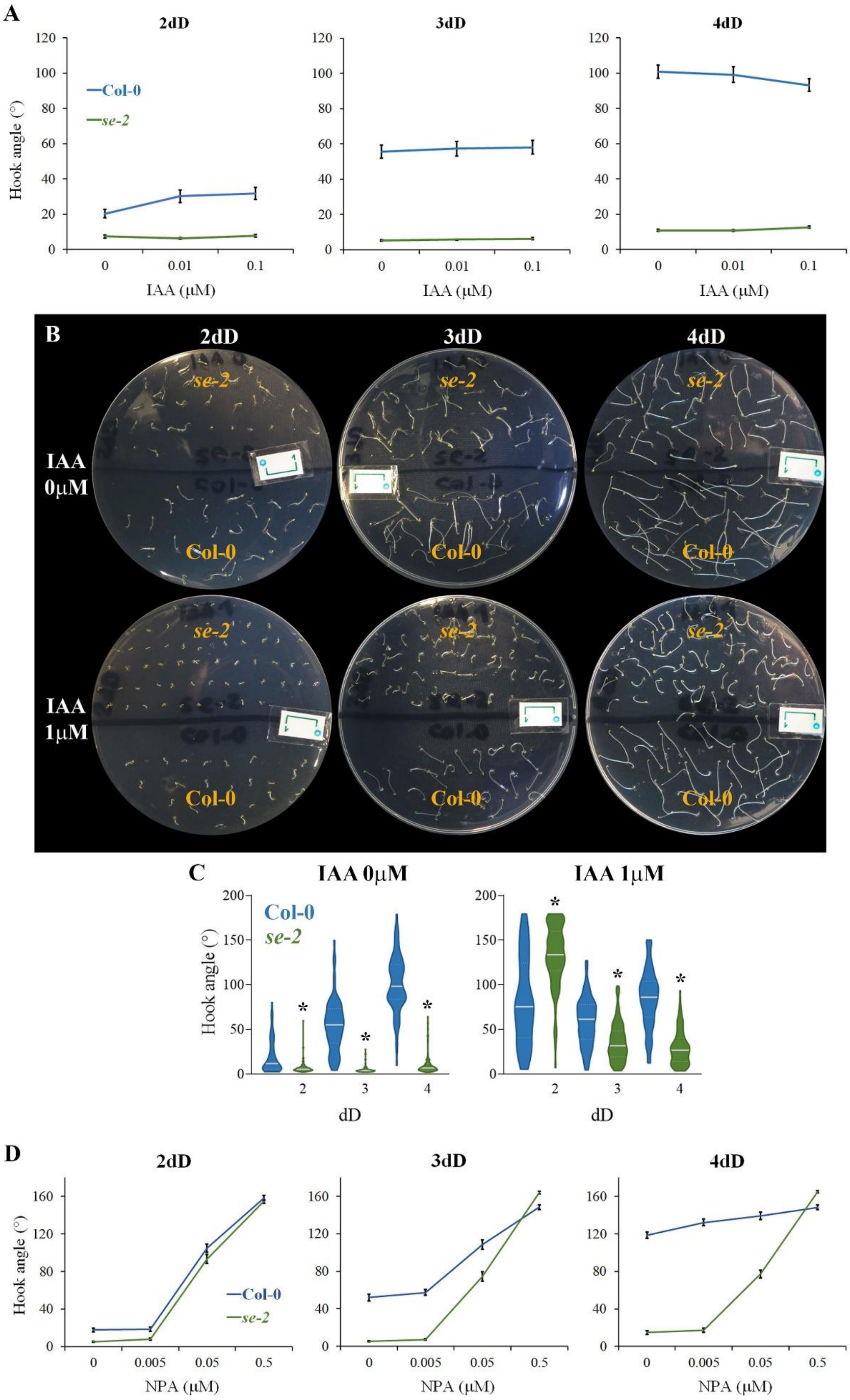
A
2dD
3dD
4dD
Hook angle (°)
Col-0
se-2
IAA (μM)
B
IAA 0μM
IAA 1μM
C
IAA 0μM
IAA 1μM
dD
D
NPA (μM)

Supplemental Figure S2. Physiological behavior of *se-2* mutants in darkness is similar to *se-1*. (A), hook angle measurements of 2, 3 and 4-days dark-grown (dD) at 22°C Col-0 and *se-2* (CS870474) seedlings. Indol acetic acid (IAA) was added to the growth medium (0.5xMS) to the final concentrations indicated, where ethanol was added to the final concentration of 0.0008% for the mock treatment (0μM IAA). Hook angle (the angle between the hypocotyl and an imaginary line between the cotyledons) was measured with ImageJ 1.49k software. Data are reported as mean ± SEM of between 24 and 49 seedlings from two biological replicates. (B), 2, 3 and 4-days dark-grown (dD) at 22°C Col-0, and *se-2* seedlings in 0.5xMS treated with 1μM IAA or mock. Seedlings were gently laid down in the medium before taking the photographs. (C), hook angle measurements of seedlings from (B). Between 21 and 53 seedlings for each sample and two biological replicates were measured. Statistically significant differences between genotypes are indicated by asterisks (t-test, $p < 0.05$). (D), NPA response assay in hooks of 2, 3 and 4-days dark-grown (dD) at 22°C Col-0 and *se-2* seedlings. The polar auxin transport inhibitor Naphthylphtalamic acid (NPA) was added to the growth medium (0.5xMS) to the final concentrations indicated. Hook angles values were measured with ImageJ 1.49k software and are represented as mean ± SEM of between 24 and 61 seedlings from two biological replicates.

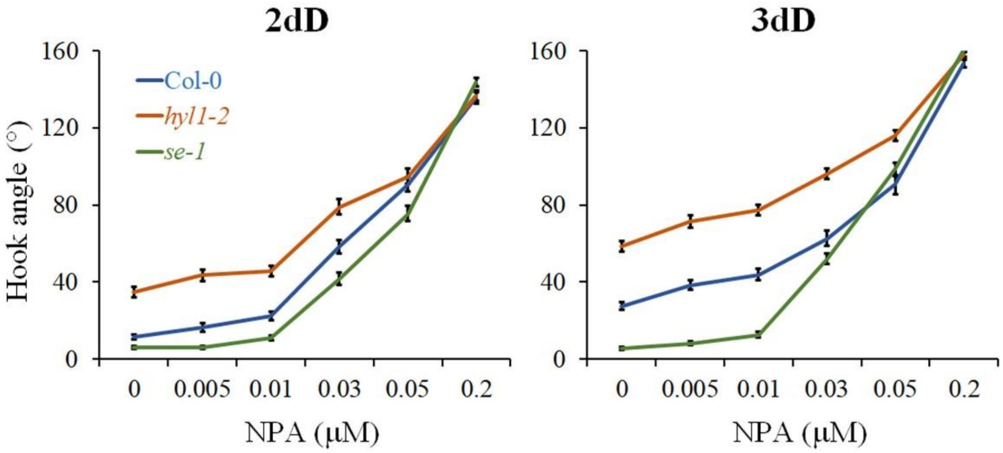

Supplemental Figure S3. NPA response assay in hooks of microprocessor mutants. Hook angle measurements of 2- and 3-days dark-grown (dD) at 22°C Col-0, *hyl1-2* and *se-1* seedlings. The polar auxin transport inhibitor Naphthylphtalamic acid (NPA) was added to the growth medium (0.5xMS) to the final concentrations indicated. Hook angles values were measured with ImageJ 1.49k software and are represented as mean ± SEM of between 35 and 54 seedlings from two biological replicates.

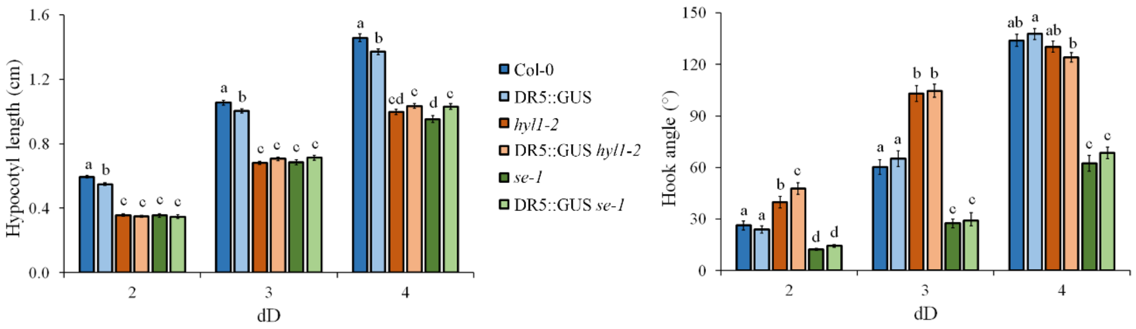

Supplemental Figure S4. Physiological behavior of DR5::GUS marker lines in darkness. Hypocotyl length and hook angle measurements of 2, 3 and 4-days dark-grown (dD) at 22°C DR5::GUS, DR5::GUS *hyl1-2* and DR5::GUS *se-1* and their corresponding genetic backgrounds. Data are reported as mean ± SEM of between 35 and 72 seedlings from two biological replicates. Statistically significant differences between groups are indicated by different letters (ANOVA and Tukey's post-test, $p < 0.05$).

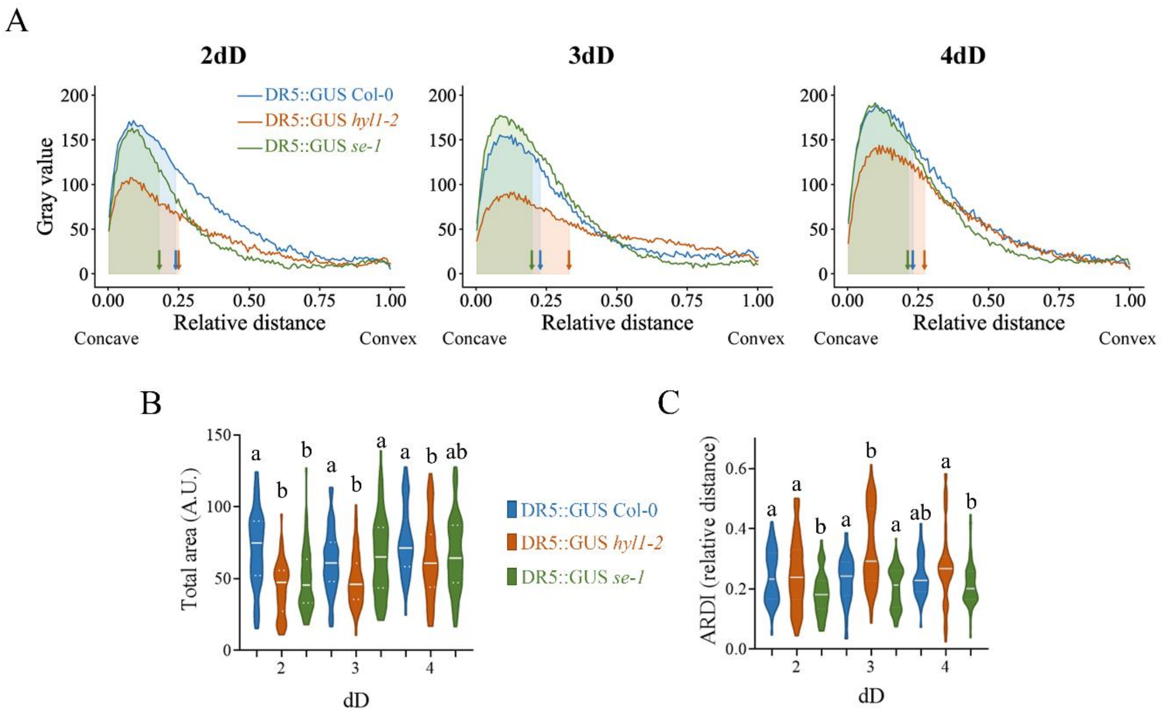

Supplemental Figure S5. Auxin differential accumulation in *hyl1-2* and *se-1* hooks. (A), gray value pattern of GUS signals along the hook of 2, 3 and 4-days dark-grown (dD), respectively, at 22°C DR5::GUS, DR5::GUS *hyl1-2* and DR5::GUS *se-1* seedlings (referred to Figure 2D). Shaded areas correspond to half of total integrated signals along the hook, where arrows indicate the relative distance for auxin response distribution index quantified in (C). (B), total auxin response area intensity from each genotype and dD, measured by signal integration. (C), ARDI (auxin response distribution index) is the relative distance shown by arrows in (A). Total area and ARDI for each genotype and dD in (B) and (C) were obtained with a home-made Python script (https://github.com/paulavacs/GUSquantification.git) from between 10 and 38 seedlings per sample from three biological replicates. Statistically significant differences between groups in (B) and (C) are indicated by different letters (one-way ANOVA and Tukey's post-test, $p < 0.05$). Statistical analysis was carried out with Infostat Software v. 2017 (Facultad de Ciencias Agropecuarias, Universidad Nacional de Córdoba, Córdoba, Argentina).

Supplemental Table S1. Statistical analysis (Figure 1A and Figure 2A).

**Table Analyzed**	**2dD hook (+IAA)**				
Two-way ANOVA	Ordinary				
Alpha	0.05				
Source of Variation	% of total variation	P value	P value summary	Significant ?	
Interaction	0.5845	0.356	ns	No	
Genotype	24.24	<0,0001	****	Yes	
Treatment with IAA	0.7678	0.0565	ns	No	
ANOVA table	SS (Type III)	DF	MS	F (DFn, DFd)	P value
Interaction	1617	4	404.3	F (4, 560) = 1,099	P=0,3560
Genotype	67073	2	33536	F (2, 560) = 91,18	P<0,0001
Treatment with IAA	2125	2	1062	F (2, 560) = 2,888	P=0,0565
Residual	205960	560	367.8		
Data summary					
Number of columns (Treatment with IAA)	3				
Number of rows (Genotype)	3				
Number of values	569				
Number of families	1				
Number of comparisons per family	36				
Alpha	0.05				
Tukey's multiple comparisons test	Predicted (LS) mean diff,	95,00% CI of diff,	Significant?	Summary	Adjusted P Value
Col-0:IAA 0 vs. Col-0:IAA 0.01	1.027	-9,573 to 11,63	No	ns	>0,9999
Col-0:IAA 0 vs. Col-0:IAA 0.1	-0.9682	-11,63 to 9,690	No	ns	>0,9999
Col-0:IAA 0 vs. *hyl1-2*:IAA 0	-19.25	-30,37 to -8,137	Yes	****	<0,0001

Col-0:IAA 0 vs. *hyl1-2*:IAA 0.01	-12.9	-23,82 to -1,974	Yes	**	0.0079
Col-0:IAA 0 vs. *hyl1-2*:IAA 0.1	-18.74	-29,49 to -7,990	Yes	****	<0,0001
Col-0:IAA 0 vs. *se-1*:IAA 0	12.73	1,338 to 24,12	Yes	*	0.0157
Col-0:IAA 0 vs. *se-1*:IAA 0.01	11.31	-0,3473 to 22,97	No	ns	0.0653
Col-0:IAA 0 vs. *se-1*:IAA 0.1	5.164	-6,276 to 16,60	No	ns	0.8956
Col-0:IAA 0.01 vs. Col-0:IAA 0.1	-1.995	-11,68 to 7,694	No	ns	0.9994
Col-0:IAA 0.01 vs. *hyl1-2*:IAA 0	-20.28	-30,47 to -10,09	Yes	****	<0,0001
Col-0:IAA 0.01 vs. *hyl1-2*:IAA 0.01	-13.92	-23,90 to -3,945	Yes	***	0.0006
Col-0:IAA 0.01 vs. *hyl1-2*:IAA 0.1	-19.77	-29,56 to -9,977	Yes	****	<0,0001
Col-0:IAA 0.01 vs. *se-1*:IAA 0	11.7	1,213 to 22,19	Yes	*	0.016
Col-0:IAA 0.01 vs. *se-1*:IAA 0.01	10.29	-0,4949 to 21,07	No	ns	0.075
Col-0:IAA 0.01 vs. *se-1*:IAA 0.1	4.137	-6,406 to 14,68	No	ns	0.9515
Col-0:IAA 0.1 vs. *hyl1-2*:IAA 0	-18.28	-28,54 to -8,034	Yes	****	<0,0001
Col-0:IAA 0.1 vs. *hyl1-2*:IAA 0.01	-11.93	-21,97 to -1,888	Yes	**	0.0073
Col-0:IAA 0.1 vs. *hyl1-2*:IAA 0.1	-17.77	-27,63 to -7,920	Yes	****	<0,0001
Col-0:IAA 0.1 vs. *se-1*:IAA 0	13.7	3,149 to 24,24	Yes	**	0.002
Col-0:IAA 0.1 vs. *se-1*:IAA 0.01	12.28	1,443 to 23,12	Yes	*	0.0133
Col-0:IAA 0.1 vs. *se-1*:IAA 0.1	6.132	-4,469 to 16,73	No	ns	0.6815
hyl1-2:IAA 0 vs. *hyl1-2*:IAA 0.01	6.357	-4,166 to 16,88	No	ns	0.6271
hyl1-2:IAA 0 vs. *hyl1-2*:IAA 0.1	0.5121	-9,835 to 10,86	No	ns	>0,9999
hyl1-2:IAA 0 vs. *se-1*:IAA 0	31.98	20,97 to 42,99	Yes	****	<0,0001
hyl1-2:IAA 0 vs. *se-1*:IAA 0.01	30.57	19,28 to 41,86	Yes	****	<0,0001
hyl1-2:IAA 0 vs. *se-1*:IAA 0.1	24.42	13,36 to 35,48	Yes	****	<0,0001
hyl1-2:IAA 0.01 vs. *hyl1-2*:IAA 0.1	-5.845	-15,98 to 4,292	No	ns	0.6852
hyl1-2:IAA 0.01 vs. *se-1*:IAA 0	25.62	14,81 to 36,44	Yes	****	<0,0001
hyl1-2:IAA 0.01 vs. *se-1*:IAA 0.01	24.21	13,11 to 35,31	Yes	****	<0,0001
hyl1-2:IAA 0.01 vs. *se-1*:IAA 0.1	18.06	7,194 to 28,93	Yes	****	<0,0001

hyl1-2:IAA 0.1 vs. *se-1*:IAA 0	31.47	20,83 to 42,11	Yes	****	<0,0001
hyl1-2:IAA 0.1 vs. *se-1*:IAA 0.01	30.05	19,12 to 40,98	Yes	****	<0,0001
hyl1-2:IAA 0.1 vs. *se-1*:IAA 0.1	23.91	13,21 to 34,60	Yes	****	<0,0001
se-1:IAA 0 vs. *se-1*:IAA 0.01	-1.414	-12,97 to 10,14	No	ns	>0,9999
se-1:IAA 0 vs. *se-1*:IAA 0.1	-7.563	-18,90 to 3,774	No	ns	0.4899
se-1:IAA 0.01 vs. *se-1*:IAA 0.1	-6.149	-17,76 to 5,460	No	ns	0.7768

**Table Analyzed**	**3dD hook (+IAA)**				
Two-way ANOVA	Ordinary				
Alpha	0.05				
Source of Variation	% of total variation	P value	P value summary	Significant ?	
Interaction	0.6303	0.0626	ns	No	
Genotype	54.64	<0,0001	****	Yes	
Treatment with IAA	0.2428	0.1779	ns	No	
ANOVA table	SS (Type III)	DF	MS	F (DFn, DFd)	P value
Interaction	4134	4	1033	F (4, 640) = 2,247	P=0,0626
Genotype	358350	2	179175	F (2, 640) = 389,6	P<0,0001
Treatment with IAA	1592	2	796.1	F (2, 640) = 1,731	P=0,1779
Residual	294333	640	459.9		
Data summary					
Number of columns (Treatment with IAA)	3				
Number of rows (Genotype)	3				
Number of values	649				
Number of families	1				
Number of comparisons per family	36				
Alpha	0.05				

Tukey's multiple comparisons test	Predicted (LS) mean diff,	95,00% CI of diff,	Significant?	Summary	Adjusted P Value
Col-0:IAA 0 vs. Col-0:IAA 0.01	5.508	-5,155 to 16,17	No	ns	0.8005
Col-0:IAA 0 vs. Col-0:IAA 0.1	5.987	-5,070 to 17,04	No	ns	0.7555
Col-0:IAA 0 vs. *hyl1-2*:IAA 0	-21.92	-33,33 to -10,51	Yes	****	<0,0001
Col-0:IAA 0 vs. *hyl1-2*:IAA 0.01	-16.76	-28,05 to -5,481	Yes	***	0.0002
Col-0:IAA 0 vs. *hyl1-2*:IAA 0.1	-17.03	-28,48 to -5,578	Yes	***	0.0002
Col-0:IAA 0 vs. *se-1*:IAA 0	42	30,64 to 53,37	Yes	****	<0,0001
Col-0:IAA 0 vs. *se-1*:IAA 0.01	42.57	31,16 to 53,97	Yes	****	<0,0001
Col-0:IAA 0 vs. *se-1*:IAA 0.1	34.5	23,44 to 45,56	Yes	****	<0,0001
Col-0:IAA 0.01 vs. Col-0:IAA 0.1	0.4791	-9,946 to 10,90	No	ns	>0,9999
Col-0:IAA 0.01 vs. *hyl1-2*:IAA 0	-27.43	-38,22 to -16,63	Yes	****	<0,0001
Col-0:IAA 0.01 vs. *hyl1-2*:IAA 0.01	-22.27	-32,93 to -11,61	Yes	****	<0,0001
Col-0:IAA 0.01 vs. *hyl1-2*:IAA 0.1	-22.54	-33,38 to -11,70	Yes	****	<0,0001
Col-0:IAA 0.01 vs. *se-1*:IAA 0	36.5	25,74 to 47,25	Yes	****	<0,0001
Col-0:IAA 0.01 vs. *se-1*:IAA 0.01	37.06	26,26 to 47,85	Yes	****	<0,0001
Col-0:IAA 0.01 vs. *se-1*:IAA 0.1	28.99	18,56 to 39,41	Yes	****	<0,0001
Col-0:IAA 0.1 vs. *hyl1-2*:IAA 0	-27.91	-39,09 to -16,72	Yes	****	<0,0001
Col-0:IAA 0.1 vs. *hyl1-2*:IAA 0.01	-22.75	-33,81 to -11,69	Yes	****	<0,0001
Col-0:IAA 0.1 vs. *hyl1-2*:IAA 0.1	-23.02	-34,25 to -11,79	Yes	****	<0,0001
Col-0:IAA 0.1 vs. *se-1*:IAA 0	36.02	24,87 to 47,16	Yes	****	<0,0001
Col-0:IAA 0.1 vs. *se-1*:IAA 0.01	36.58	25,39 to 47,76	Yes	****	<0,0001
Col-0:IAA 0.1 vs. *se-1*:IAA 0.1	28.51	17,68 to 39,34	Yes	****	<0,0001
hyl1-2:IAA 0 vs. *hyl1-2*:IAA 0.01	5.157	-6,251 to 16,56	No	ns	0.8951
hyl1-2:IAA 0 vs. *hyl1-2*:IAA 0.1	4.89	-6,685 to 16,47	No	ns	0.9268
hyl1-2:IAA 0 vs. *se-1*:IAA 0	63.92	52,43 to 75,41	Yes	****	<0,0001
hyl1-2:IAA 0 vs. *se-1*:IAA 0.01	64.49	52,95 to 76,02	Yes	****	<0,0001

hyl1-2:IAA 0 vs. *se-1*:IAA 0.1	56.42	45,23 to 67,60	Yes	****	<0,0001
hyl1-2:IAA 0.01 vs. *hyl1-2*:IAA 0.1	-0.2668	-11,72 to 11,19	No	ns	>0,9999
hyl1-2:IAA 0.01 vs. *se-1*:IAA 0	58.77	47,40 to 70,13	Yes	****	<0,0001
hyl1-2:IAA 0.01 vs. *se-1*:IAA 0.01	59.33	47,92 to 70,74	Yes	****	<0,0001
hyl1-2:IAA 0.01 vs. *se-1*:IAA 0.1	51.26	40,20 to 62,32	Yes	****	<0,0001
hyl1-2:IAA 0.1 vs. *se-1*:IAA 0	59.03	47,50 to 70,57	Yes	****	<0,0001
hyl1-2:IAA 0.1 vs. *se-1*:IAA 0.01	59.6	48,02 to 71,17	Yes	****	<0,0001
hyl1-2:IAA 0.1 vs. *se-1*:IAA 0.1	51.53	40,30 to 62,76	Yes	****	<0,0001
se-1:IAA 0 vs. *se-1*:IAA 0.01	0.5627	-10,93 to 12,05	No	ns	>0,9999
se-1:IAA 0 vs. *se-1*:IAA 0.1	-7.505	-18,65 to 3,636	No	ns	0.4765
se-1:IAA 0.01 vs. *se-1*:IAA 0.1	-8.068	-19,25 to 3,117	No	ns	0.3778

**Table Analyzed**	**4dD hook (+IAA)**				
Two-way ANOVA	Ordinary				
Alpha	0.05				
Source of Variation	% of total variation	P value	P value summary	Significant ?	
Interaction	0.3983	0.1109	ns	No	
Genotype	61.58	<0,0001	****	Yes	
Treatment with IAA	1.612	<0,0001	****	Yes	
ANOVA table	SS (Type III)	DF	MS	F (DFn, DFd)	P value
Interaction	5067	4	1267	F (4, 684) = 1,887	P=0,1109
Genotype	783423	2	391712	F (2, 684) = 583,5	P<0,0001
Treatment with IAA	20509	2	10255	F (2, 684) = 15,28	P<0,0001
Residual	459144	684	671.3		
Data summary					
Number of columns (Treatment with IAA)	3				

Number of rows (Genotype)	3				
Number of values	693				
Number of families	1				
Number of comparisons per family	36				
Alpha	0.05				
Tukey's multiple comparisons test	Predicted (LS) mean diff,	95,00% CI of diff,	Significant?	Summary	Adjusted P Value
Col-0:IAA 0 vs. Col-0:IAA 0.01	5.665	-7,754 to 19,08	No	ns	0.9272
Col-0:IAA 0 vs. Col-0:IAA 0.1	16.68	2,987 to 30,38	Yes	**	0.0051
Col-0:IAA 0 vs. *hyl1-2*:IAA 0	-16.84	-31,09 to -2,596	Yes	**	0.0077
Col-0:IAA 0 vs. *hyl1-2*:IAA 0.01	-9.211	-22,84 to 4,418	No	ns	0.472
Col-0:IAA 0 vs. *hyl1-2*:IAA 0.1	2.791	-10,90 to 16,49	No	ns	0.9994
Col-0:IAA 0 vs. *se-1*:IAA 0	67.76	53,51 to 82,01	Yes	****	<0,0001
Col-0:IAA 0 vs. *se-1*:IAA 0.01	73.84	60,18 to 87,50	Yes	****	<0,0001
Col-0:IAA 0 vs. *se-1*:IAA 0.1	73.16	58,50 to 87,82	Yes	****	<0,0001
Col-0:IAA 0.01 vs. Col-0:IAA 0.1	11.02	-1,119 to 23,15	No	ns	0.11
Col-0:IAA 0.01 vs. *hyl1-2*:IAA 0	-22.51	-35,27 to -9,753	Yes	****	<0,0001
Col-0:IAA 0.01 vs. *hyl1-2*:IAA 0.01	-14.88	-26,94 to -2,815	Yes	**	0.0043
Col-0:IAA 0.01 vs. *hyl1-2*:IAA 0.1	-2.874	-15,01 to 9,261	No	ns	0.9982
Col-0:IAA 0.01 vs. *se-1*:IAA 0	62.1	49,34 to 74,85	Yes	****	<0,0001
Col-0:IAA 0.01 vs. *se-1*:IAA 0.01	68.17	56,08 to 80,27	Yes	****	<0,0001
Col-0:IAA 0.01 vs. *se-1*:IAA 0.1	67.5	54,28 to 80,71	Yes	****	<0,0001
Col-0:IAA 0.1 vs. *hyl1-2*:IAA 0	-33.53	-46,57 to -20,48	Yes	****	<0,0001
Col-0:IAA 0.1 vs. *hyl1-2*:IAA 0.01	-25.89	-38,26 to -13,52	Yes	****	<0,0001
Col-0:IAA 0.1 vs. *hyl1-2*:IAA 0.1	-13.89	-26,33 to -1,450	Yes	*	0.0158
Col-0:IAA 0.1 vs. *se-1*:IAA 0	51.08	38,03 to 64,13	Yes	****	<0,0001
Col-0:IAA 0.1 vs. *se-1*:IAA 0.01	57.16	44,75 to 69,56	Yes	****	<0,0001

Col-0:IAA 0.1 vs. *se-1*:IAA 0.1	56.48	42,98 to 69,98	Yes	****	<0,0001
hyl1-2:IAA 0 vs. *hyl1-2*:IAA 0.01	7.634	-5,344 to 20,61	No	ns	0.6619
hyl1-2:IAA 0 vs. *hyl1-2*:IAA 0.1	19.64	6,589 to 32,68	Yes	***	0.0001
hyl1-2:IAA 0 vs. *se-1*:IAA 0	84.61	70,98 to 98,23	Yes	****	<0,0001
hyl1-2:IAA 0 vs. *se-1*:IAA 0.01	90.68	77,67 to 103,7	Yes	****	<0,0001
hyl1-2:IAA 0 vs. *se-1*:IAA 0.1	90.01	75,95 to 104,1	Yes	****	<0,0001
hyl1-2:IAA 0.01 vs. *hyl1-2*:IAA 0.1	12	-0,3657 to 24,37	No	ns	0.0652
hyl1-2:IAA 0.01 vs. *se-1*:IAA 0	76.97	63,99 to 89,95	Yes	****	<0,0001
hyl1-2:IAA 0.01 vs. *se-1*:IAA 0.01	83.05	70,72 to 95,38	Yes	****	<0,0001
hyl1-2:IAA 0.01 vs. *se-1*:IAA 0.1	82.37	68,94 to 95,80	Yes	****	<0,0001
hyl1-2:IAA 0.1 vs. *se-1*:IAA 0	64.97	51,92 to 78,02	Yes	****	<0,0001
hyl1-2:IAA 0.1 vs. *se-1*:IAA 0.01	71.05	58,65 to 83,45	Yes	****	<0,0001
hyl1-2:IAA 0.1 vs. *se-1*:IAA 0.1	70.37	56,87 to 83,87	Yes	****	<0,0001
se-1:IAA 0 vs. *se-1*:IAA 0.01	6.077	-6,935 to 19,09	No	ns	0.8764
se-1:IAA 0 vs. *se-1*:IAA 0.1	5.399	-8,662 to 19,46	No	ns	0.9575
se-1:IAA 0.01 vs. *se-1*:IAA 0.1	-0.6788	-14,14 to 12,79	No	ns	>0,9999

**Table Analyzed**	**2dD hook (+NPA)**				
Two-way ANOVA	Ordinary				
Alpha	0.05				
Source of Variation	% of total variation	P value	P value summary	Significant ?	
Interaction	3.005	<0,0001	****	Yes	
Genotype	1.077	<0,0001	****	Yes	
Treatment with NPA	84.84	<0,0001	****	Yes	
ANOVA table	SS (Type III)	DF	MS	F (DFn, DFd)	P value
Interaction	148883	6	24814	F (6, 1033) = 48,59	P<0,0001

Genotype	53364	2	26682	F (2, 1033) = 52,25	P<0,0001
Treatment with NPA	4202981	3	1400994	F (3, 1033) = 2744	P<0,0001
Residual	527480	1033	510.6		
Data summary					
Number of columns (Treatment with NPA)	4				
Number of rows (Genotype)	3				
Number of values	1045				
Number of families	1				
Number of comparisons per family	66				
Alpha	0.05				
Tukey's multiple comparisons test	Predicted (LS) mean diff,	95,00% CI of diff,	Significant?	Summary	Adjusted P Value
Col-0:NPA 0 vs. Col-0:NPA 0.005	-1.228	-12,42 to 9,963	No	ns	>0,9999
Col-0:NPA 0 vs. Col-0:NPA 0.05	-120.8	-131,6 to -110,0	Yes	****	<0,0001
Col-0:NPA 0 vs. Col-0:NPA 0.5	-156.4	-167,3 to -145,4	Yes	****	<0,0001
Col-0:NPA 0 vs. *hyl1-2*:NPA 0	-34.49	-45,88 to -23,09	Yes	****	<0,0001
Col-0:NPA 0 vs. *hyl1-2*:NPA 0.005	-41.37	-52,91 to -29,83	Yes	****	<0,0001
Col-0:NPA 0 vs. *hyl1-2*:NPA 0.05	-122.5	-134,0 to -111,1	Yes	****	<0,0001
Col-0:NPA 0 vs. *hyl1-2*:NPA 0.5	-140.4	-151,5 to -129,2	Yes	****	<0,0001
Col-0:NPA 0 vs. *se-1*:NPA 0	4.405	-7,527 to 16,34	No	ns	0.9883
Col-0:NPA 0 vs. *se-1*:NPA 0.005	5.019	-6,562 to 16,60	No	ns	0.9597
Col-0:NPA 0 vs. *se-1*:NPA 0.05	-133.8	-144,8 to -122,7	Yes	****	<0,0001
Col-0:NPA 0 vs. *se-1*:NPA 0.5	-151.3	-162,0 to -140,5	Yes	****	<0,0001
Col-0:NPA 0.005 vs. Col-0:NPA 0.05	-119.6	-130,3 to -108,8	Yes	****	<0,0001
Col-0:NPA 0.005 vs. Col-0:NPA 0.5	-155.1	-166,1 to -144,2	Yes	****	<0,0001
Col-0:NPA 0.005 vs. *hyl1-2*:NPA 0	-33.26	-44,62 to -21,90	Yes	****	<0,0001
Col-0:NPA 0.005 vs. *hyl1-2*:NPA 0.005	-40.14	-51,66 to -28,63	Yes	****	<0,0001

Col-0:NPA 0.005 vs. *hyl1-2*:NPA 0.05	-121.3	-132,7 to -109,9	Yes	****	<0,0001
Col-0:NPA 0.005 vs. *hyl1-2*:NPA 0.5	-139.1	-150,3 to -128,0	Yes	****	<0,0001
Col-0:NPA 0.005 vs. *se-1*:NPA 0	5.633	-6,269 to 17,53	No	ns	0.926
Col-0:NPA 0.005 vs. *se-1*:NPA 0.005	6.247	-5,303 to 17,80	No	ns	0.8332
Col-0:NPA 0.005 vs. *se-1*:NPA 0.05	-132.5	-143,5 to -121,6	Yes	****	<0,0001
Col-0:NPA 0.005 vs. *se-1*:NPA 0.5	-150	-160,8 to -139,3	Yes	****	<0,0001
Col-0:NPA 0.05 vs. Col-0:NPA 0.5	-35.55	-46,07 to -25,02	Yes	****	<0,0001
Col-0:NPA 0.05 vs. *hyl1-2*:NPA 0	86.33	75,38 to 97,29	Yes	****	<0,0001
Col-0:NPA 0.05 vs. *hyl1-2*:NPA 0.005	79.45	68,34 to 90,56	Yes	****	<0,0001
Col-0:NPA 0.05 vs. *hyl1-2*:NPA 0.05	-1.713	-12,74 to 9,318	No	ns	>0,9999
Col-0:NPA 0.05 vs. *hyl1-2*:NPA 0.5	-19.55	-30,26 to -8,839	Yes	****	<0,0001
Col-0:NPA 0.05 vs. *se-1*:NPA 0	125.2	113,7 to 136,7	Yes	****	<0,0001
Col-0:NPA 0.05 vs. *se-1*:NPA 0.005	125.8	114,7 to 137,0	Yes	****	<0,0001
Col-0:NPA 0.05 vs. *se-1*:NPA 0.05	-12.94	-23,50 to -2,384	Yes	**	0.0037
Col-0:NPA 0.05 vs. *se-1*:NPA 0.5	-30.44	-40,75 to -20,12	Yes	****	<0,0001
Col-0:NPA 0.5 vs. *hyl1-2*:NPA 0	121.9	110,7 to 133,0	Yes	****	<0,0001
Col-0:NPA 0.5 vs. *hyl1-2*:NPA 0.005	115	103,7 to 126,3	Yes	****	<0,0001
Col-0:NPA 0.5 vs. *hyl1-2*:NPA 0.05	33.83	22,60 to 45,06	Yes	****	<0,0001
Col-0:NPA 0.5 vs. *hyl1-2*:NPA 0.5	15.99	5,075 to 26,91	Yes	***	0.0001
Col-0:NPA 0.5 vs. *se-1*:NPA 0	160.8	149,1 to 172,5	Yes	****	<0,0001
Col-0:NPA 0.5 vs. *se-1*:NPA 0.005	161.4	150,0 to 172,7	Yes	****	<0,0001
Col-0:NPA 0.5 vs. *se-1*:NPA 0.05	22.6	11,83 to 33,37	Yes	****	<0,0001
Col-0:NPA 0.5 vs. *se-1*:NPA 0.5	5.109	-5,421 to 15,64	No	ns	0.913
hyl1-2:NPA 0 vs. *hyl1-2*:NPA 0.005	-6.886	-18,59 to 4,821	No	ns	0.7423
hyl1-2:NPA 0 vs. *hyl1-2*:NPA 0.05	-88.04	-99,68 to -76,41	Yes	****	<0,0001
hyl1-2:NPA 0 vs. *hyl1-2*:NPA 0.5	-105.9	-117,2 to -94,55	Yes	****	<0,0001
hyl1-2:NPA 0 vs. *se-1*:NPA 0	38.89	26,80 to 50,98	Yes	****	<0,0001

hyl1-2:NPA 0 vs. *se-1*:NPA 0.005	39.51	27,76 to 51,25	Yes	****	<0,0001
hyl1-2:NPA 0 vs. *se-1*:NPA 0.05	-99.27	-110,5 to -88,09	Yes	****	<0,0001
hyl1-2:NPA 0 vs. *se-1*:NPA 0.5	-116.8	-127,7 to -105,8	Yes	****	<0,0001
hyl1-2:NPA 0.005 vs. *hyl1-2*:NPA 0.05	-81.16	-92,94 to -69,38	Yes	****	<0,0001
hyl1-2:NPA 0.005 vs. *hyl1-2*:NPA 0.5	-99	-110,5 to -87,52	Yes	****	<0,0001
hyl1-2:NPA 0.005 vs. *se-1*:NPA 0	45.78	33,54 to 58,01	Yes	****	<0,0001
hyl1-2:NPA 0.005 vs. *se-1*:NPA 0.005	46.39	34,50 to 58,28	Yes	****	<0,0001
hyl1-2:NPA 0.005 vs. *se-1*:NPA 0.05	-92.39	-103,7 to -81,05	Yes	****	<0,0001
hyl1-2:NPA 0.005 vs. *se-1*:NPA 0.5	-109.9	-121,0 to -98,77	Yes	****	<0,0001
hyl1-2:NPA 0.05 vs. *hyl1-2*:NPA 0.5	-17.84	-29,24 to -6,435	Yes	****	<0,0001
hyl1-2:NPA 0.05 vs. *se-1*:NPA 0	126.9	114,8 to 139,1	Yes	****	<0,0001
hyl1-2:NPA 0.05 vs. *se-1*:NPA 0.005	127.5	115,7 to 139,4	Yes	****	<0,0001
hyl1-2:NPA 0.05 vs. *se-1*:NPA 0.05	-11.23	-22,49 to 0,02925	No	ns	0.0513
hyl1-2:NPA 0.05 vs. *se-1*:NPA 0.5	-28.72	-39,75 to -17,69	Yes	****	<0,0001
hyl1-2:NPA 0.5 vs. *se-1*:NPA 0	144.8	132,9 to 156,6	Yes	****	<0,0001
hyl1-2:NPA 0.5 vs. *se-1*:NPA 0.005	145.4	133,9 to 156,9	Yes	****	<0,0001
hyl1-2:NPA 0.5 vs. *se-1*:NPA 0.05	6.608	-4,339 to 17,56	No	ns	0.7089
hyl1-2:NPA 0.5 vs. *se-1*:NPA 0.5	-10.89	-21,60 to -0,1733	Yes	*	0.0424
se-1:NPA 0 vs. *se-1*:NPA 0.005	0.6147	-11,66 to 12,88	No	ns	>0,9999
se-1:NPA 0 vs. *se-1*:NPA 0.05	-138.2	-149,9 to -126,4	Yes	****	<0,0001
se-1:NPA 0 vs. *se-1*:NPA 0.5	-155.7	-167,2 to -144,1	Yes	****	<0,0001
se-1:NPA 0.005 vs. *se-1*:NPA 0.05	-138.8	-150,2 to -127,4	Yes	****	<0,0001
se-1:NPA 0.005 vs. *se-1*:NPA 0.5	-156.3	-167,4 to -145,1	Yes	****	<0,0001
se-1:NPA 0.05 vs. *se-1*:NPA 0.5	-17.49	-28,05 to -6,936	Yes	****	<0,0001

**Table Analyzed**	**3dD hook (+NPA)**				
Two-way ANOVA	Ordinary				
Alpha	0.05				
Source of Variation	% of total variation	P value	P value summary	Significant ?	
Interaction	7.049	<0,0001	****	Yes	
Genotype	6.212	<0,0001	****	Yes	
Treatment with NPA	69.28	<0,0001	****	Yes	
ANOVA table	SS (Type III)	DF	MS	F (DFn, DFd)	P value
Interaction	267416	6	44569	F (6, 1069) = 81,08	P<0,0001
Genotype	235695	2	117848	F (2, 1069) = 214,4	P<0,0001
Treatment with NPA	2628415	3	876138	F (3, 1069) = 1594	P<0,0001
Residual	587623	1069	549.7		
Data summary					
Number of columns (Treatment with NPA)	4				
Number of rows (Genotype)	3				
Number of values	1081				
Number of families	1				
Number of comparisons per family	66				
Alpha	0.05				
Tukey's multiple comparisons test	Predicted (LS) mean diff,	95,00% CI of diff,	Significant?	Summary	Adjusted P Value
Col-0:NPA 0 vs. Col-0:NPA 0.005	-8.467	-19,96 to 3,027	No	ns	0.3982
Col-0:NPA 0 vs. Col-0:NPA 0.05	-77.33	-88,09 to -66,57	Yes	****	<0,0001
Col-0:NPA 0 vs. Col-0:NPA 0.5	-113.4	-125,4 to -101,4	Yes	****	<0,0001
Col-0:NPA 0 vs. *hyl1-2*:NPA 0	-36.53	-48,05 to -25,00	Yes	****	<0,0001

Col-0:NPA 0 vs. *hyl1-2*:NPA 0.005	-45.18	-57,53 to -32,83	Yes	****	<0,0001
Col-0:NPA 0 vs. *hyl1-2*:NPA 0.05	-85.14	-96,96 to -73,33	Yes	****	<0,0001
Col-0:NPA 0 vs. *hyl1-2*:NPA 0.5	-120.8	-132,7 to -108,8	Yes	****	<0,0001
Col-0:NPA 0 vs. *se-1*:NPA 0	33.74	22,00 to 45,49	Yes	****	<0,0001
Col-0:NPA 0 vs. *se-1*:NPA 0.005	35.14	23,65 to 46,64	Yes	****	<0,0001
Col-0:NPA 0 vs. *se-1*:NPA 0.05	-89.66	-101,3 to -78,01	Yes	****	<0,0001
Col-0:NPA 0 vs. *se-1*:NPA 0.5	-119.5	-130,8 to -108,1	Yes	****	<0,0001
Col-0:NPA 0.005 vs. Col-0:NPA 0.05	-68.87	-79,31 to -58,42	Yes	****	<0,0001
Col-0:NPA 0.005 vs. Col-0:NPA 0.5	-104.9	-116,7 to -93,22	Yes	****	<0,0001
Col-0:NPA 0.005 vs. *hyl1-2*:NPA 0	-28.06	-39,29 to -16,83	Yes	****	<0,0001
Col-0:NPA 0.005 vs. *hyl1-2*:NPA 0.005	-36.71	-48,79 to -24,64	Yes	****	<0,0001
Col-0:NPA 0.005 vs. *hyl1-2*:NPA 0.05	-76.68	-88,21 to -65,15	Yes	****	<0,0001
Col-0:NPA 0.005 vs. *hyl1-2*:NPA 0.5	-112.3	-123,9 to -100,6	Yes	****	<0,0001
Col-0:NPA 0.005 vs. *se-1*:NPA 0	42.21	30,75 to 53,67	Yes	****	<0,0001
Col-0:NPA 0.005 vs. *se-1*:NPA 0.005	43.61	32,41 to 54,81	Yes	****	<0,0001
Col-0:NPA 0.005 vs. *se-1*:NPA 0.05	-81.19	-92,55 to -69,83	Yes	****	<0,0001
Col-0:NPA 0.005 vs. *se-1*:NPA 0.5	-111	-122,1 to -99,91	Yes	****	<0,0001
Col-0:NPA 0.05 vs. Col-0:NPA 0.5	-36.07	-47,07 to -25,07	Yes	****	<0,0001
Col-0:NPA 0.05 vs. *hyl1-2*:NPA 0	40.8	30,32 to 51,28	Yes	****	<0,0001
Col-0:NPA 0.05 vs. *hyl1-2*:NPA 0.005	32.15	20,77 to 43,53	Yes	****	<0,0001
Col-0:NPA 0.05 vs. *hyl1-2*:NPA 0.05	-7.812	-18,61 to 2,987	No	ns	0.4281
Col-0:NPA 0.05 vs. *hyl1-2*:NPA 0.5	-43.42	-54,34 to -32,50	Yes	****	<0,0001
Col-0:NPA 0.05 vs. *se-1*:NPA 0	111.1	100,4 to 121,8	Yes	****	<0,0001
Col-0:NPA 0.05 vs. *se-1*:NPA 0.005	112.5	102,0 to 122,9	Yes	****	<0,0001
Col-0:NPA 0.05 vs. *se-1*:NPA 0.05	-12.32	-22,94 to -1,708	Yes	**	0.0083
Col-0:NPA 0.05 vs. *se-1*:NPA 0.5	-42.13	-52,46 to -31,81	Yes	****	<0,0001
Col-0:NPA 0.5 vs. *hyl1-2*:NPA 0	76.88	65,13 to 88,63	Yes	****	<0,0001

Col-0:NPA 0.5 vs. *hyl1-2*:NPA 0.005	68.22	55,67 to 80,78	Yes	****	<0,0001
Col-0:NPA 0.5 vs. *hyl1-2*:NPA 0.05	28.26	16,22 to 40,29	Yes	****	<0,0001
Col-0:NPA 0.5 vs. *hyl1-2*:NPA 0.5	-7.351	-19,49 to 4,792	No	ns	0.7052
Col-0:NPA 0.5 vs. *se-1*:NPA 0	147.1	135,2 to 159,1	Yes	****	<0,0001
Col-0:NPA 0.5 vs. *se-1*:NPA 0.005	148.5	136,8 to 160,3	Yes	****	<0,0001
Col-0:NPA 0.5 vs. *se-1*:NPA 0.05	23.75	11,88 to 35,62	Yes	****	<0,0001
Col-0:NPA 0.5 vs. *se-1*:NPA 0.5	-6.063	-17,67 to 5,548	No	ns	0.8636
hyl1-2:NPA 0 vs. *hyl1-2*:NPA 0.005	-8.652	-20,75 to 3,450	No	ns	0.4475
hyl1-2:NPA 0 vs. *hyl1-2*:NPA 0.05	-48.62	-60,18 to -37,06	Yes	****	<0,0001
hyl1-2:NPA 0 vs. *hyl1-2*:NPA 0.5	-84.23	-95,90 to -72,56	Yes	****	<0,0001
hyl1-2:NPA 0 vs. *se-1*:NPA 0	70.27	58,78 to 81,76	Yes	****	<0,0001
hyl1-2:NPA 0 vs. *se-1*:NPA 0.005	71.67	60,44 to 82,90	Yes	****	<0,0001
hyl1-2:NPA 0 vs. *se-1*:NPA 0.05	-53.13	-64,51 to -41,74	Yes	****	<0,0001
hyl1-2:NPA 0 vs. *se-1*:NPA 0.5	-82.94	-94,06 to -71,82	Yes	****	<0,0001
hyl1-2:NPA 0.005 vs. *hyl1-2*:NPA 0.05	-39.96	-52,34 to -27,58	Yes	****	<0,0001
hyl1-2:NPA 0.005 vs. *hyl1-2*:NPA 0.5	-75.57	-88,06 to -63,09	Yes	****	<0,0001
hyl1-2:NPA 0.005 vs. *se-1*:NPA 0	78.92	66,61 to 91,24	Yes	****	<0,0001
hyl1-2:NPA 0.005 vs. *se-1*:NPA 0.005	80.32	68,25 to 92,40	Yes	****	<0,0001
hyl1-2:NPA 0.005 vs. *se-1*:NPA 0.05	-44.48	-56,70 to -32,26	Yes	****	<0,0001
hyl1-2:NPA 0.005 vs. *se-1*:NPA 0.5	-74.29	-86,25 to -62,32	Yes	****	<0,0001
hyl1-2:NPA 0.05 vs. *hyl1-2*:NPA 0.5	-35.61	-47,57 to -23,65	Yes	****	<0,0001
hyl1-2:NPA 0.05 vs. *se-1*:NPA 0	118.9	107,1 to 130,7	Yes	****	<0,0001
hyl1-2:NPA 0.05 vs. *se-1*:NPA 0.005	120.3	108,8 to 131,8	Yes	****	<0,0001
hyl1-2:NPA 0.05 vs. *se-1*:NPA 0.05	-4.511	-16,19 to 7,170	No	ns	0.9832
hyl1-2:NPA 0.05 vs. *se-1*:NPA 0.5	-34.32	-45,74 to -22,90	Yes	****	<0,0001
hyl1-2:NPA 0.5 vs. *se-1*:NPA 0	154.5	142,6 to 166,4	Yes	****	<0,0001
hyl1-2:NPA 0.5 vs. *se-1*:NPA 0.005	155.9	144,3 to 167,5	Yes	****	<0,0001

hyl1-2:NPA 0.5 vs. *se-1*:NPA 0.05	31.1	19,31 to 42,89	Yes	****	<0,0001
hyl1-2:NPA 0.5 vs. *se-1*:NPA 0.5	1.288	-10,24 to 12,82	No	ns	>0,9999
se-1:NPA 0 vs. *se-1*:NPA 0.005	1.399	-10,06 to 12,86	No	ns	>0,9999
se-1:NPA 0 vs. *se-1*:NPA 0.05	-123.4	-135,0 to -111,8	Yes	****	<0,0001
se-1:NPA 0 vs. *se-1*:NPA 0.5	-153.2	-164,6 to -141,9	Yes	****	<0,0001
se-1:NPA 0.005 vs. *se-1*:NPA 0.05	-124.8	-136,2 to -113,4	Yes	****	<0,0001
se-1:NPA 0.005 vs. *se-1*:NPA 0.5	-154.6	-165,7 to -143,5	Yes	****	<0,0001
se-1:NPA 0.05 vs. *se-1*:NPA 0.5	-29.81	-41,05 to -18,57	Yes	****	<0,0001

**Table Analyzed**	**4dD hook (+NPA)**				
Two-way ANOVA	Ordinary				
Alpha	0.05				
Source of Variation	% of total variation	P value	P value summary	Significant ?	
Interaction	12.34	<0,0001	****	Yes	
Genotype	16.86	<0,0001	****	Yes	
Treatment with NPA	39.87	<0,0001	****	Yes	
ANOVA table	SS (Type III)	DF	MS	F (DFn, DFd)	P value
Interaction	272164	6	45361	F (6, 1106) = 76,79	P<0,0001
Genotype	371917	2	185958	F (2, 1106) = 314,8	P<0,0001
Treatment with NPA	879539	3	293180	F (3, 1106) = 496,3	P<0,0001
Residual	653292	1106	590.7		
Data summary					
Number of columns (Treatment with NPA)	4				
Number of rows (Genotype)	3				
Number of values	1118				
Number of families	1				

Number of comparisons per family	66				
Alpha	0.05				
Tukey's multiple comparisons test	Predicted (LS) mean diff,	95,00% CI of diff,	Significant?	Summary	Adjusted P Value
Col-0:NPA 0 vs. Col-0:NPA 0.005	-10.33	-22,40 to 1,751	No	ns	0.1812
Col-0:NPA 0 vs. Col-0:NPA 0.05	-38.35	-49,87 to -26,83	Yes	****	<0,0001
Col-0:NPA 0 vs. Col-0:NPA 0.5	-55.98	-68,00 to -43,96	Yes	****	<0,0001
Col-0:NPA 0 vs. *hyl1-2*:NPA 0	-25.75	-38,20 to -13,30	Yes	****	<0,0001
Col-0:NPA 0 vs. *hyl1-2*:NPA 0.005	-29.32	-42,02 to -16,61	Yes	****	<0,0001
Col-0:NPA 0 vs. *hyl1-2*:NPA 0.05	-47.98	-60,30 to -35,67	Yes	****	<0,0001
Col-0:NPA 0 vs. *hyl1-2*:NPA 0.5	-59	-71,44 to -46,55	Yes	****	<0,0001
Col-0:NPA 0 vs. *se-1*:NPA 0	54.93	42,06 to 67,80	Yes	****	<0,0001
Col-0:NPA 0 vs. *se-1*:NPA 0.005	51.31	39,51 to 63,12	Yes	****	<0,0001
Col-0:NPA 0 vs. *se-1*:NPA 0.05	-28.39	-40,24 to -16,54	Yes	****	<0,0001
Col-0:NPA 0 vs. *se-1*:NPA 0.5	-61.5	-73,91 to -49,09	Yes	****	<0,0001
Col-0:NPA 0.005 vs. Col-0:NPA 0.05	-28.02	-38,80 to -17,24	Yes	****	<0,0001
Col-0:NPA 0.005 vs. Col-0:NPA 0.5	-45.65	-56,97 to -34,34	Yes	****	<0,0001
Col-0:NPA 0.005 vs. *hyl1-2*:NPA 0	-15.42	-27,18 to -3,661	Yes	**	0.0011
Col-0:NPA 0.005 vs. *hyl1-2*:NPA 0.005	-18.99	-31,03 to -6,957	Yes	****	<0,0001
Col-0:NPA 0.005 vs. *hyl1-2*:NPA 0.05	-37.66	-49,28 to -26,04	Yes	****	<0,0001
Col-0:NPA 0.005 vs. *hyl1-2*:NPA 0.5	-48.67	-60,43 to -36,91	Yes	****	<0,0001
Col-0:NPA 0.005 vs. *se-1*:NPA 0	65.26	53,05 to 77,47	Yes	****	<0,0001
Col-0:NPA 0.005 vs. *se-1*:NPA 0.005	61.64	50,56 to 72,72	Yes	****	<0,0001
Col-0:NPA 0.005 vs. *se-1*:NPA 0.05	-18.06	-29,19 to -6,934	Yes	****	<0,0001
Col-0:NPA 0.005 vs. *se-1*:NPA 0.5	-51.17	-62,90 to -39,45	Yes	****	<0,0001
Col-0:NPA 0.05 vs. Col-0:NPA 0.5	-17.63	-28,35 to -6,914	Yes	****	<0,0001
Col-0:NPA 0.05 vs. *hyl1-2*:NPA 0	12.6	1,411 to 23,79	Yes	*	0.0126

Col-0:NPA 0.05 vs. *hyl1-2*:NPA 0.005	9.03	-2,447 to 20,51	No	ns	0.2941
Col-0:NPA 0.05 vs. *hyl1-2*:NPA 0.05	-9.636	-20,68 to 1,405	No	ns	0.1577
Col-0:NPA 0.05 vs. *hyl1-2*:NPA 0.5	-20.65	-31,84 to -9,461	Yes	****	<0,0001
Col-0:NPA 0.05 vs. *se-1*:NPA 0	93.28	81,62 to 104,9	Yes	****	<0,0001
Col-0:NPA 0.05 vs. *se-1*:NPA 0.005	89.66	79,19 to 100,1	Yes	****	<0,0001
Col-0:NPA 0.05 vs. *se-1*:NPA 0.05	9.959	-0,5635 to 20,48	No	ns	0.0836
Col-0:NPA 0.05 vs. *se-1*:NPA 0.5	-23.15	-34,30 to -12,00	Yes	****	<0,0001
Col-0:NPA 0.5 vs. *hyl1-2*:NPA 0	30.23	18,53 to 41,94	Yes	****	<0,0001
Col-0:NPA 0.5 vs. *hyl1-2*:NPA 0.005	26.66	14,68 to 38,64	Yes	****	<0,0001
Col-0:NPA 0.5 vs. *hyl1-2*:NPA 0.05	7.996	-3,570 to 19,56	No	ns	0.503
Col-0:NPA 0.5 vs. *hyl1-2*:NPA 0.5	-3.018	-14,72 to 8,688	No	ns	0.9995
Col-0:NPA 0.5 vs. *se-1*:NPA 0	110.9	98,75 to 123,1	Yes	****	<0,0001
Col-0:NPA 0.5 vs. *se-1*:NPA 0.005	107.3	96,27 to 118,3	Yes	****	<0,0001
Col-0:NPA 0.5 vs. *se-1*:NPA 0.05	27.59	16,52 to 38,66	Yes	****	<0,0001
Col-0:NPA 0.5 vs. *se-1*:NPA 0.5	-5.517	-17,19 to 6,152	No	ns	0.9265
hyl1-2:NPA 0 vs. *hyl1-2*:NPA 0.005	-3.57	-15,97 to 8,834	No	ns	0.9987
hyl1-2:NPA 0 vs. *hyl1-2*:NPA 0.05	-22.24	-34,24 to -10,23	Yes	****	<0,0001
hyl1-2:NPA 0 vs. *hyl1-2*:NPA 0.5	-33.25	-45,39 to -21,11	Yes	****	<0,0001
hyl1-2:NPA 0 vs. *se-1*:NPA 0	80.68	68,10 to 93,26	Yes	****	<0,0001
hyl1-2:NPA 0 vs. *se-1*:NPA 0.005	77.06	65,58 to 88,54	Yes	****	<0,0001
hyl1-2:NPA 0 vs. *se-1*:NPA 0.05	-2.641	-14,17 to 8,887	No	ns	0.9998
hyl1-2:NPA 0 vs. *se-1*:NPA 0.5	-35.75	-47,85 to -23,65	Yes	****	<0,0001
hyl1-2:NPA 0.005 vs. *hyl1-2*:NPA 0.05	-18.67	-30,94 to -6,394	Yes	****	<0,0001
hyl1-2:NPA 0.005 vs. *hyl1-2*:NPA 0.5	-29.68	-42,08 to -17,27	Yes	****	<0,0001
hyl1-2:NPA 0.005 vs. *se-1*:NPA 0	84.25	71,42 to 97,08	Yes	****	<0,0001
hyl1-2:NPA 0.005 vs. *se-1*:NPA 0.005	80.63	68,87 to 92,39	Yes	****	<0,0001
hyl1-2:NPA 0.005 vs. *se-1*:NPA 0.05	0.9292	-10,88 to 12,74	No	ns	>0,9999

hyl1-2:NPA 0.005 vs. *se-1*:NPA 0.5	-32.18	-44,55 to -19,81	Yes	****	<0,0001
hyl1-2:NPA 0.05 vs. *hyl1-2*:NPA 0.5	-11.01	-23,02 to 0,9894	No	ns	0.1083
hyl1-2:NPA 0.05 vs. *se-1*:NPA 0	102.9	90,47 to 115,4	Yes	****	<0,0001
hyl1-2:NPA 0.05 vs. *se-1*:NPA 0.005	99.3	87,96 to 110,6	Yes	****	<0,0001
hyl1-2:NPA 0.05 vs. *se-1*:NPA 0.05	19.59	8,210 to 30,98	Yes	****	<0,0001
hyl1-2:NPA 0.05 vs. *se-1*:NPA 0.5	-13.51	-25,48 to -1,546	Yes	*	0.0122
hyl1-2:NPA 0.5 vs. *se-1*:NPA 0	113.9	101,4 to 126,5	Yes	****	<0,0001
hyl1-2:NPA 0.5 vs. *se-1*:NPA 0.005	110.3	98,83 to 121,8	Yes	****	<0,0001
hyl1-2:NPA 0.5 vs. *se-1*:NPA 0.05	30.61	19,08 to 42,14	Yes	****	<0,0001
hyl1-2:NPA 0.5 vs. *se-1*:NPA 0.5	-2.5	-14,60 to 9,604	No	ns	>0,9999
se-1:NPA 0 vs. *se-1*:NPA 0.005	-3.617	-15,56 to 8,324	No	ns	0.9979
se-1:NPA 0 vs. *se-1*:NPA 0.05	-83.32	-95,31 to -71,33	Yes	****	<0,0001
se-1:NPA 0 vs. *se-1*:NPA 0.5	-116.4	-129,0 to -103,9	Yes	****	<0,0001
se-1:NPA 0.005 vs. *se-1*:NPA 0.05	-79.7	-90,54 to -68,87	Yes	****	<0,0001
se-1:NPA 0.005 vs. *se-1*:NPA 0.5	-112.8	-124,3 to -101,4	Yes	****	<0,0001
se-1:NPA 0.05 vs. *se-1*:NPA 0.5	-33.11	-44,60 to -21,62	Yes	****	<0,0001

Supplemental Table S2. Primers used in RT-qPCR (Figure 1B and Figure 1C).

Gene	AGI number		Sequence
TAA1	AT1G70560	Fwd	GGAGACTTGTAAGAGCGAGTCC
		Rev	ACTTGGGAAGAGTGAAAGCATC
TAR2	AT4G24670	Fwd	GTGTCAGACAGTTGTGGGAATG
		Rev	CAACGTTGAGAGACGAAATCAG
YUC4	AT5G11320	Fwd	GTTTACTGGACCGGTTGTTC
		Rev	AGATCCAAGCTGACCTCCATAC
YUC6	AT5G25620	Fwd	CACAAGTGTTGGAAACAAGACG
		Rev	AATTCCCACCACAATCACTCTC
IAA2	AT3G23030	Fwd	GAAGAATCTACACCTCCTACCAAAA
		Rev	CACGTAGCTCACACTGTTGTTG
IAA7	AT3G23050	Fwd	AAGCTACCAGGATCTTTCTGATGC
		Rev	ATTCCTTGTGCTCCATAGTTTCCC
IAA14	AT4G14550	Fwd	AAATTCAGGACCCATGAGAGGC
		Rev	GCACATTAGCATGAAGAGGATGG
IAA19	AT3G15540	Fwd	GAGCATGGATGGTGTGCCTTAT
		Rev	TTCGCAGTTGTCACCATCTTTC
ARF7	AT5G20730	Fwd	GCGGCTAAAACAAGAACTCG
		Rev	CGCCTCCATCTAAACCGTAA
ARF19	AT1G19220	Fwd	TCCAGTGCTGCAATCAGTTC
		Rev	CCTCCACCATTCATGATTCC
SAUR16	AT4G38860	Fwd	TGCTACGACGAGGAAGGTCT
		Rev	ACCTTGTACGCTTTTCGCCT
SAUR50	AT4G34760	Fwd	AAGATTGCCTTCCGCTTGAC
		Rev	CGGCTCGTTGTAAGAGAGATTG
PP2AA3	AT1G13320	Fwd	CCTGCGGTAATAACTGCATCT
		Rev	CTTCACTTAGCTCCACCAAGCA
